# Understanding Malaria Treatment Adherence in Rwanda: Implications for Artemisinin Resistance

**DOI:** 10.4269/ajtmh.25-0061

**Published:** 2025-10-28

**Authors:** Pierre Gashema, Aileen Jordan, Eric Saramba, Neeva Wernsman Young, Patrick Gad Iradukunda, Corine Karema, Jean Baptiste Mazarati, Jonathan J. Juliano, Jeffrey A. Bailey, Kristin Banek

**Affiliations:** ^1^ College of Medicine and Veterinary Medicine, University of Edinburgh, Edinburgh, United Kingdom;; ^2^ Center for Genomic Biology, INES-Ruhengeri, Ruhengeri, Rwanda;; ^3^ College of Medicine and Health Sciences, University of Rwanda, Kigali, Rwanda;; ^4^ Center for Computational Molecular Biology, Brown University, Providence, Rhode Island;; ^5^ Repolicy Research Centre, Kigali, Rwanda;; ^6^ Drug Department, Rwanda Food and Drugs Authority, Kigali, Rwanda;; ^7^ Quality Equity Health Care, Kigali, Rwanda;; ^8^ Division of Infectious Diseases, School of Medicine, University of North Carolina, Chapel Hill, North Carolina;; ^9^ Institute for Global Health and Infectious Diseases, University of North Carolina, Chapel Hill, North Carolina;; ^10^ Department of Pathology, Brown University, Providence, Rhode Island;; ^11^ Department of Epidemiology, Tulane University Celia Scott Weatherhead School of Public Health and Tropical Medicine, New Orleans, Louisiana

## Abstract

Prompt diagnosis and effective treatment are key malaria interventions that rely on community knowledge and adherence to treatment. With the emergence of artemisinin resistance in Rwanda, ensuring optimal malaria treatment practices within communities is essential. This study examined malaria knowledge, attitudes, and practices among febrile patients at government clinics to identify factors influencing malaria treatment practices. A cross-sectional study was conducted in six health facilities in moderate- to high-malaria-transmission areas of Rwanda. Patients or caregivers of children with fevers were enrolled and interviewed using semistructured questionnaires. From December 2023 to February 2024, 406 participants were enrolled; 71% (*n* = 289/406) of participants owned insecticide-treated nets, and 51% (*n* = 205/406) received indoor residual spraying. Malaria knowledge was high among respondents, with 81% (*n* = 329/406) correctly identifying symptoms, 72% (*n* = 291/406) understanding transmission modes, and 74.6% (*n* = 303/406) aware of effective control measures. However, of the 44.3% (*n* = 180/406) who received malaria treatment in the last 6 months, only 46% (*n* = 83/180) completed the appropriate 3-day medication course; 37% (*n* = 66/180) stopped within 2 days, and 11% (*n* = 19/180) went over 3 days. Furthermore, 27% (*n* = 109/406) took antimalarials for fever; the majority (54%; *n* = 49/109) received medication from drug outlets/pharmacies. Although knowledge and attitudes toward malaria treatment were high, adherence was poor, thereby exacerbating the risk of developing resistance. Effective interventions are urgently needed to improve antimalarial adherence, particularly in sub-Saharan African countries with documented antimalarial resistance.

## INTRODUCTION

Despite significant gains, malaria remains a major public health concern, with the WHO reporting 249 million cases in 2022, an increase of 5 million cases observed since 2021.^1^ The vast majority of malaria-related mortality (95.4%) remains in the WHO Africa region.^1^ Over the past two decades, Rwanda has seen significant variation in malaria incidence. During the period 2005–2010, malaria incidence in Rwanda decreased from 162 per 1,000 population to 62 per 1,000 population, with the dramatic decrease linked to multiple malaria control interventions (i.e., improved treatment and vector control)^2^ as well as the adoption of artemisinin-based combination therapies (ACTs) as the first-line malaria treatment in 2006.^3^ These interventions resulted in a decrease of over 50% in associated admissions and deaths at health facilities during this time period.^3^ However, between 2011 and 2017, the Rwanda National Malaria Control Program (NMCP) reported an eightfold increase in malaria cases countrywide from 45 to 486 per 1,000 population.^4^ This resurgence was likely driven by multiple factors both related and unrelated to specific malaria control interventions.^5^

To combat this resurgence, the Rwanda Malaria Strategic Plan 2020–2024 aimed to reduce mortality and morbidity related to malaria by using key malaria control interventions.^6^ Other than vector control interventions, malaria control depends on effective case management, which requires timely, accurate diagnosis and importantly, appropriate treatment with effective antimalarials.^2^ In part, the NMCP instituted the expansion of malaria treatment from central to peripheral levels, including community-based case management with approximately 60,000 community health workers (CHWs; two per village referred to as binômes).^5^ Community health workers are trained through the complementary programs of integrated community case management of fever and home-based management of fever to use malaria rapid diagnostic tests, dispense ACTs, and refer severe cases.^7^ The integration of CHWs helped the Rwanda Ministry of Health to respond promptly to malaria cases, and according to the WHO, 55% of malaria cases were diagnosed and treated by CHWs in Rwanda by the end of 2022 compared with 15% in 2016.^8^ However, artemisinin partial resistance (ArtR) threatens this new progress. Rwanda was the first African nation to document ArtR in cases from 2014,^9^ and now, ArtR-conferring mutations are prevalent nationwide, complicating the future of malaria treatment in Rwanda.^10^^–^^12^

The overarching goal of the national malaria strategic plan is to interrupt malaria transmission.^13^ Community education and knowledge about malaria are important for effective resource utilization and will likely become more important as malaria transmission decreases, resulting in less daily contact with the disease.^14^ Rwanda has assessed community knowledge and practice of malaria interventions; however, the data are limited. In Ruhuha, one of the endemic areas in eastern Rwanda, a survey conducted in 2015 showed that 80.1%, 91.4%, and 87.3% of community members had good knowledge, attitudes, and practices (KAP), respectively, toward malaria interventions.^15^ In 2015, Asingizwe et al.^16^ evaluated Rwandan community readiness on pre-elimination in the same area and revealed that 90.7% of individuals were knowledgeable about malaria elimination strategies. In neighboring Uganda, local knowledge of malaria among patients was shown to drive appropriate case management and use of antimalarials.^17^ To update and expand understanding of community-level knowledge and factors influencing malaria treatment adherence in Rwanda, we conducted a malaria KAP survey among patients seeking care for fever in six regions of Rwanda to identify factors associated with malaria treatment practices.

## MATERIALS AND METHODS

### Study context.

**Figure 1.**
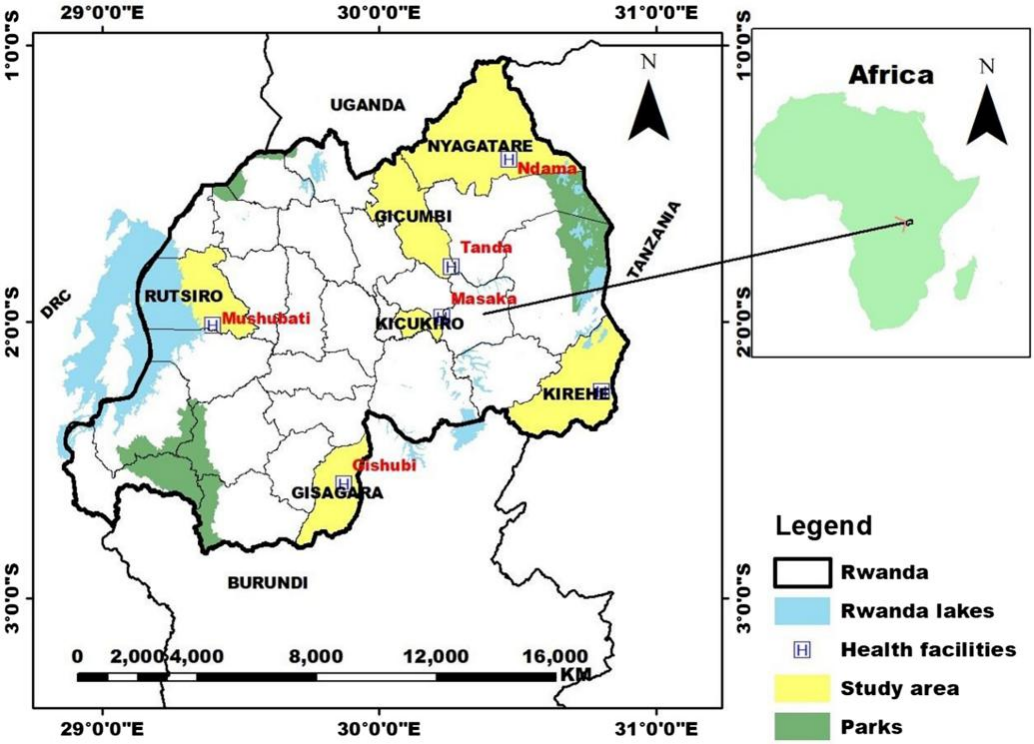
Map of the study area. Health facilities (red) and their corresponding districts (yellow) where the study was conducted are shown. Kirehe, Gisagara, and Nyagatare are considered high-malaria-transmission areas, whereas Kicukiro, Mushubati, and Gicumbi are classified as moderate-transmission areas. Except for Kicukiro District, the others are rural areas.

This study was conducted in six governmental health facilities (Ndama and Mahama in Eastern Province, Mushubati in Western Province, Tanda in Northern Province, Gishubi in Southern Province, and Masaka in Kigali City) across rural and urban areas in Rwanda. We deliberately selected these health facilities based on case load, endemicity, and geographical context (urban/rural), ensuring representation from all provinces (Figure 1).^18^ A convenience sampling approach was used, where febrile patients were recruited after their consultations. Eligible participants provided informed consent and completed a structured questionnaire, with each interview lasting approximately 30 minutes. After each interview, the next patient exiting the consultation room was screened, ensuring that they had fever but no severe disease or other conditions preventing participation. This sequential process ensured systematic recruitment while reducing selection bias.

### Study design and sample size.

A health facility-based cross-sectional study was conducted to assess the KAP regarding malaria treatment among patients seeking treatment of fever. The aim was to identify factors that influence malaria treatment practices. The intended sample size of 400 fever patients was calculated using the Yamane formula based on an estimated population of 13 million for Rwanda, a margin of error (*e*) of 0.05.^19^

### Data collection.

A standard KAP questionnaire was developed based on previous Rwanda Malaria Indicator Surveys and the National Malaria Survey in Sierra Leone.^18^^,^^20^ The questionnaire included demographic characteristics of the participants, knowledge of participants of malaria disease (symptoms and transmission), preventive measures for malaria, malaria treatment, and attitudes toward existing control measures and treatment practices as shown in the questionnaire in Supplemental Appendix 1. All questionnaires and consent forms were first developed in English; they were later translated into Kinyarwanda and back to English to ensure that meaning was retained. Each participant was assigned a unique identification number based on the name of the health facility.

### Knowledge, attitudes, and practices scores and outcome definitions.

Quality scores were derived to assess participants’ KAP. The score for each participant was obtained by summing the scores of all items in the three sections and then, calculating the total as a percentage of the maximum possible score. The scaled quality score is calculated using the formula scaled quality score (obtained score − minimum possible score divided by maximum possible score − minimum possible score) × 100%.^21^ Treatment adherence was defined as taking antimalarial treatment for 3 days.

## STATISTICAL ANALYSES

Responses were collected using paper forms, and all data were entered and validated in a Microsoft Excel spreadsheet (Microsoft Corp, Redmond, WA). Analyses were conducted in SPSS v. 2023 (IBM Corp., Armonk, NY) and Stata 16 (StataCorp, College Station, TX). Basic descriptive statistics were used to calculate the frequencies and proportions of demographic characteristics of participants and the KAP indicators. Logistic regression was used to estimate the crude (bivariate) and adjusted (multivariate) odds ratios and their 95% CIs to assess the association between participant characteristics and KAP indicators and the two adherence outcomes (diagnosed malaria treatment and fever treatment). Covariates were assessed for correlation using the Pearson correlation coefficient; none were found to be strongly correlated (*r* ≥0.8). All covariates were included in multivariable analyses regardless of *P*-values. Covariates with small cell sizes or low frequencies were excluded from multivariable analysis to avoid bias and unreliable estimates. Associations were considered significant if the *P*-value was <0.05.

## RESULTS

### Participant characteristics.

A total of 406 patients/caregivers were enrolled in the study from December 2023 to February 2024. Of those enrolled, 17% (*n* = 69/406) of them were from Gishubi Health Center, 16% (*n* = 66/406) were from Mahama, 17% (*n* = 70/406) were from Masaka, 16% (*n* = 65/406) were from Mushubati, 18% (*n* = 68/406) were from Ndama, and 17% (*n* = 68/406) were from Tanda. Demographic characteristics are summarized in Table 1. Over half (56%; *n* = 228/406) of the participants were female, and 80% (*n* = 324/406) lived in rural areas. Half of all respondents attended primary school, and 28.8% (*n* = 88/406) had the secondary level of education and above. The mean age of the study participants was 29 years old. The majority of participants (59.6%; *n* = 242/406) were farmers. The primary religion of participants was Catholic (40.1%; *n* = 163/406).

**Table 1:** Participant characteristics (*N* = 406)

Variable and Categories	*N* (%)
Location	
Rural	324/406 (79.8%)
Urban	82/406 (20.2%)
Health facility	
Gishubi	69/406 (17.0%)
Mahama	66/406 (16.3%)
Masaka	70/406 (17.2%)
Mushubati	65/406 (16.0%)
Ndama	68/406 (16.7%)
Tanda	68/406 (16.7%)
Sex	
Male	178/406 (43.8%)
Female	228/406 (56.2%)
Age categories, years	
Younger than 15	65/406 (16%)
15 and older	341/406 (84.0%)
Occupation	
Farmer	242/406 (59.6%)
Local leader	13/406 (3.2%)
Small trade	50/406 (12.3%)
Vocational work	101/406 (25.0%)
Religion	
Catholic	163/406 (40.1%)
Protestant	133/406 (32.8%)
Adventist	55/406 (13.5%)
Evangelical	55/406 (13.5%)
Highest level of education	
No formal education	85/406 (20.9%)
Primary	204/406 (50.2%)
Secondary and above	88/406 (28.8%)

### Knowledge of malaria symptoms, transmission, control, prevention, and treatment.

**Figure 2.**
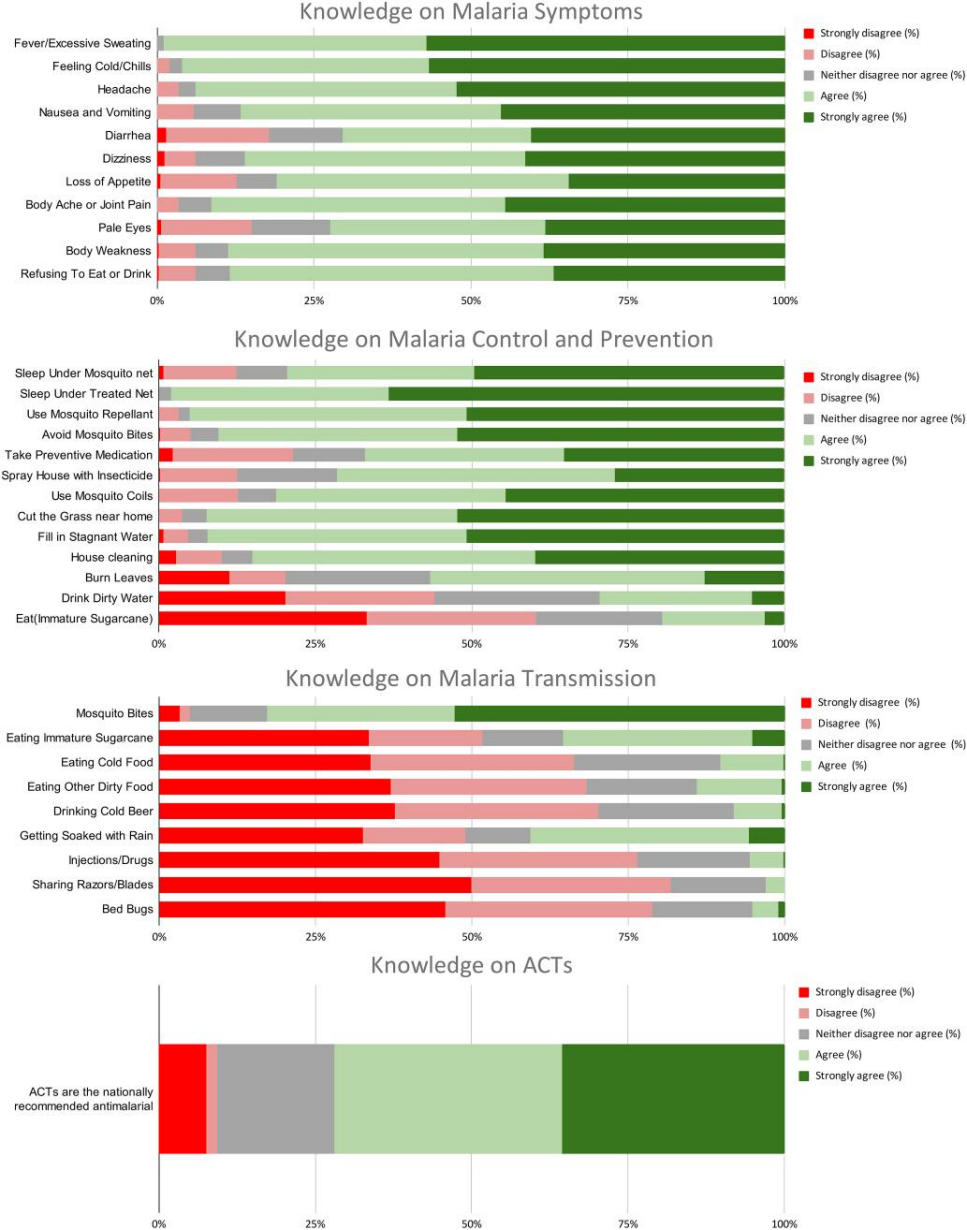
Details of the knowledge on malaria symptoms, transmission, control, prevention, and treatment. Red indicates strongly disagree, pink indicates disagree, gray indicates neutral, light green indicates agree, and dark green indicates strongly agree. ACT = artemisinin-based combination therapy.

The study revealed that participants demonstrated a combined score of 81% (*n* = 329/406) for understanding malaria signs and symptoms, with 99% (*n* = 402/406) recognizing fever as a key symptom and 70.4% (*n* = 286/406) identifying diarrhea as a possible indicator. Similarly, participants achieved a combined score of 72% (*n* = 292/406) for awareness of malaria transmission, with all 100% (*n* = 406/406) of participants correctly identifying mosquito bites as the primary mode of transmission. However, significant misconceptions were noted, with rain exposure, immature sugarcane consumption, and bed bugs noted as causes of malaria by 40% (*n* = 162/406), 35% (*n* = 142/406), and 52% (*n* = 211/406), respectively. Concerning treatment, 72% (*n* = 292/406) of respondents were aware that ACTs are the recommended treatment for malaria, although 18.7% (*n* = 76/406) expressed uncertainty about this. Preventive practices were encouraging, with 98% (*n* = 389/406) agreeing that sleeping under treated nets is the most effective prevention strategy, 71.4% (*n* = 290/406) endorsing indoor residual spraying, and 67% (*n* = 272/406) supporting presumptive treatment. Overall, participants demonstrated a strong knowledge and practice of malaria prevention and control, achieving an average score of 74.6% (*n* = 303/406), although targeted education is needed to address persistent misconceptions and improve treatment awareness (Figure 2).

### Attitudes on malaria control methods and treatment.

**Figure 3.**
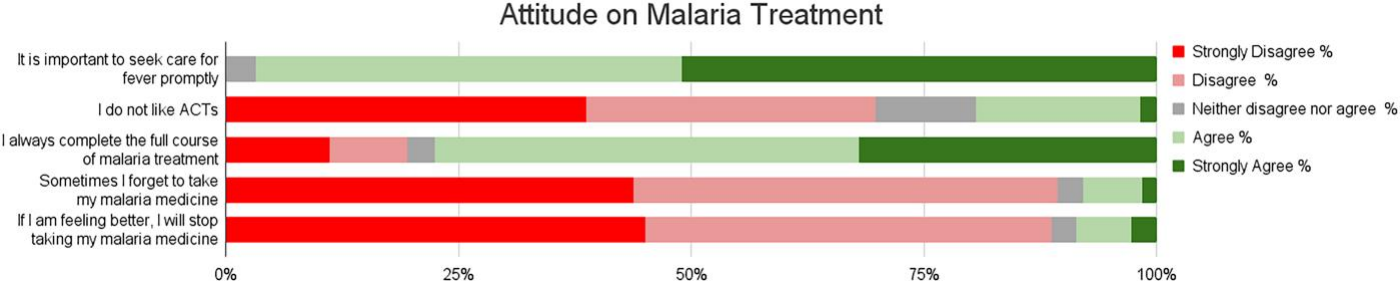
Malaria treatment attitudes and reported practices. Respondents’ degree of agreement with statements with regard to malaria treatment are shown. Red indicates strongly disagree, pink indicates disagree, gray indicates neutral, light green indicates agree, and dark green indicates strongly agree. ACT = artemisinin-based combination therapy.

Participants demonstrated mixed attitudes toward malaria control methods as shown in Supplemental Appendix 2. Although 36.2% (*n* = 147/406) expressed a positive view of chemicals used for indoor residual spraying, 26.6% (*n* = 113/406) believed that spraying alone negates the need for other preventive measures. Additionally, 52.9% (*n* = 215/406) agreed that sleeping under mosquito nets is sufficient to prevent malaria, and 76.3% (*n* = 310/406) had a positive perception of the nets’ safety. Concerning net usability, 40.6% (*n* = 165/406) approved of their use because they are provided for free, although a small proportion (5.6%; *n* = 23/406) reported using nets for agricultural purposes. Regarding malaria treatment, most participants (96.8%; *n* = 393/406) sought care promptly when experiencing fever, and 68.7% (*n* = 279/406) had a favorable attitude toward ACTs. However, 20% (*n* = 81/406) expressed a dislike for ACTs, and 8.6% (*n* = 35/406) admitted to stopping treatment prematurely when they felt better (Figure 3).

### Malaria treatment practice.

#### Adherence to malaria treatment.

Of the 406 participants interviewed, 44.3% (*n* = 180/406) had been diagnosed and treated for malaria in the last 6 months (diagnosed malaria). The ACT artemether-lumefantrine was used as malaria treatment for all patients (Table 2). Most study participants had been treated at the community level by CHWs (71.6%; *n* = 129/180). Fewer received care at health care structures, such as health centers (15.0%; *n* = 27/180) or outlets/pharmacies (11.1%; *n* = 20/180), or through self-medication (2.2%; *n* = 4/180). Only 46.1% (*n* = 83/180) of the study participants diagnosed with malaria took the full 3-day course of antimalarials. Additionally, 40.4% (*n* = 73/180) of respondents took medication for less than the recommended period (<3 days), 10.5% (*n* = 19/180) took medication for 4 or more days, and for 2.7% (*n* = 5/180), treatment duration was unknown.

**Table 2:** Adherence to malaria treatment (*N* = 406)

Variable	Diagnosed Malaria (*n* = 180), %	Fever (*n* = 109), %
Took any antimalarial in the last 6 months		
Yes	180/406 (44.3%)	109/406 (26.8%)
No	226/406 (55.7%)	292/406 (71.9%)
Unknown	0/406 (0.0%)	5/406 (1.2%)
Antimalarial type		
Artemether-lumefantrine	180/180 (100%)	109/109 (100%)
Source of antimalarial		
Health center	27/180 (15.0%)	2/109 (1.8%)
Outlet/pharmacy	20/180 (11.1%)	59/109 (54.1%)
Community health worker	129/180 (71.6%)	42/109 (38.5%)
Self-treatment	4/180 (2.2%)	6/109 (5.5%)
Duration of treatment, days		
1	7/180 (3.8%)	1/109 (0.9%)
2	66/180 (36.6%)	26/109 (23.8%)
3	83/180 (46.1%)	73/109 (66.9%)
4+	19/180 (10.5%)	9/109 (8.2%)
Unknown	5/180 (2.7%)	0/109 (0.0%)

A quarter of participants (26.8%; *n* = 109/406) also reported taking an antimalarial for fever (without confirmatory diagnosis). The most common source of antimalarial drugs for fever was outlets/pharmacies, accounting for 54.1% (*n* = 59/109) (Table 2). Two thirds of participants (66.9%; *n* = 73/109) took the medication as prescribed for 3 days, whereas 0.9% (*n* = 1/109), 23.8% (*n* = 26/109), and 8.2% (*n* = 9/109) took the medication for 1, 2, and 4 or more days, respectively.

#### Factors influencing adherence to malaria treatment.

Factors associated with adherence to malaria treatment are presented in Table 3. Overall, adherence was associated with gender, education, occupation, location (urban), religion, and study site (health facility). In an adjusted multivariate analysis, only occupation and religion were found to be significantly associated with adherence to treatment among individuals diagnosed with malaria (Table 3). Vocational workers, including drivers, were less likely to be adherent compared with farmers (adjusted odds ratio [aOR] = 0.19; 95% CI = 0.05–0.72; *P* = 0.015). Likewise, the adjusted odds of treatment adherence were significantly lower in participants who are Adventists compared with Catholics (aOR = 0.10; 95% CI = 0.02–0.49; *P* = 0.004). Females had twice the adjusted odds of adherence to artemether-lumefantrine compared with males (aOR = 2.01; 95% CI = 0.95–4.24; *P* = 0.067), but the result was not statistically significant. Having a history of usually completing treatment, stopping medications when feeling better, or forgetting to take malaria medication was not associated with treatment adherence. However, participants who reported disliking ACTs were less likely to be adherent to treatment; however, the finding was no longer significant after adjusting for other factors.

**Table 3:** Factors associated with adherence to the treatment of malaria

Variable	Yes	No	Unadjusted			Adjusted		
			cOR	95% CI	*P*-Value	aOR	95% CI	*P*-Value
Age, years								
Younger than 15	6 (33.3)	12 (66.8)	–	–	Reference	–	–	Reference
15 and older	77 (47.5)	85 (52.5)	1.81	0.65–5.06	0.257	0.47	0.09–2.52	0.378
Gender								
Male	40 (40.8)	58 (59.2)	–	–	Reference	–	–	Reference
Female	43 (52.4)	39 (47.6)	1.60	0.88–2.89	0.12	2.01	0.95–4.24	0.067
Occupation								
Farmer	56 (48.3)	60 (51.7)	–	–	Reference	–	–	Reference
Local leader	6 (75.0)	2 (25.0)	3.21	0.62–16.59	0.163	3.08	0.40–23.62	0.275
Small trade	15 (57.7)	11 (42.3)	1.46	0.62–3.45	0.387	2.44	0.0.84–9.16	0.092
Vocational work	6 (20.0)	24 (80.0)	0.26	0.10–0.70	**0.008**	0.19	0.05–0.72	**0.015**
Religion								
Catholic	34 (46.0)	40 (54.1)	–	–	Reference	–	–	Reference
Protestant	33 (47.8)	36 (52.17)	1.08	0.56–2.08	0.822	0.74	0.30–1.79	0.508
Adventist	6 (28.6)	15 (71.4)	0.47	0.16–1.35	0.16	0.10	0.02–0.49	**0.004**
Evangelical	10 (62.5)	6 (37.5)	1.96	0.64–5.95	0.23	2.14	0.45–10.70	0.332
Education								
None	21 (47.7)	23 (52.3)	–	–	Reference	–	–	Reference
Primary	36 (42.4)	49 (57.7)	0.80	0.39–1.67	0.56	0.74	0.27–2.02	0.559
Secondary and above	26 (50.1)	25 (49.0)	1.13	0.50–2.55	0.750	1.00	0.29–3.49	0.989
Location								
Rural	78 (49.1)	81 (50.9)	–	–	Reference	–	–	Reference
Urban	5 (23.1)	16 (76.2)	0.32	0.11–0.92	**0.036**	0.47	0.08–273	0.401
Facility								
Ndama	26 (50.0)	26 (50.0)	–	–	Reference	–	–	Reference
Mahama	4 (44.4)	5 (55.6)	0.80	0.19–3.32	0.759	1.00	0.166–6.11	0.992
Gishubi	5 (17.2)	24 (82.8)	0.21	0.07–0.63	**0.005**	1.05	0.10–10.795	0.966
Masaka*	0 (0.0)	13 (100.0)	–	–	–	1	–	–
Tanda	15 (55.6)	12 (44.4)	1.25	0.49–3.18	0.639	1.65	0.48–6.11	0.420
Mushubati	33 (64.0)	17 (34.0)	1.94	0.87–4.31	0.104	6.12	0.75–49.73	0.09
ACT knowledge								
No	47 (45.2)	57 (54.8)	–	–	Reference	–	–	Reference
Yes	36 (47.4)	40 (52.6)	0.91	0.50–1.65	0.770	2.41	0.53–11.71	0.246
ACT dislike								
No	6 (21.4)	22 (78.6)	–	–	Reference	–	–	Reference
Yes	77 (50.7)	75 (49.3)	0.26	0.10–0.69	**0.007**	0.20	0.04–1.06	0.06
Forgot to take ACT								
No	8 (72.7)	3 (27.3)	–	–	Reference	–	–	Reference
Yes	89 (52.7)	80 (47.3)	0.41	0.10–1.62	0.48	0.79	0.12–5.31	0.817
Stopped taking ACT when better								
No	11 (73.3)	4 (26.7)	–	–	Reference	–	–	–
Yes	86 (52.1)	79 (47.9)	0.39	0.12–1.29	0.125	0.41	0.08–1.99	0.273
Usually completes treatment								
No	54 (42.9)	72 (57.1)	–	–	Reference	–	–	Reference
Yes	29 (54.7)	24 (45.3)	0.62	0.32–1.18	0.150	0.89	0.25–3.08	0.858
Net use								
No	81 (45.8)	96 (54.2)	–	–	Reference	–	–	–
Yes	2 (66.7)	1 (33.3)	0.42	0.03–4.73	0.480	0.54	0.02–13.04	0.706
Treatment source								
Government sources/health center	10 (37.0)	17 (73.0)	–	–	Reference	–	–	Reference
Pharmacy/outlet	5 (25.0)	15 (75.0)	0.56	0.15–2.03	0.380	0.92	0.19–4.44	0.922
CHWs	65 (50.4)	64 (49.6)	1.72	0.73–4.05	0.210	1.85	0.58–5.90	0.297
Self-treatment	3 (75.0)	1 (25.0)	5.09	0.46–55.80	0.180	6.27	0.36–109.6	0.209

ACT = artemisinin-based combination therapy; aOR = adjusted odds ratio; CHW = community health worker; cOR = crude odds ratio.

* No patients reported adhering (3 days) to malaria treatment in Masaka.

Bold *P*-values indicate statistical significance.

#### Factors influencing adherence to fever treatment.

In an adjusted multivariate analysis, only religion was found to be significantly associated with adherence to fever treatment (Table 4). Protestants had four times the adjusted odds of adhering to fever treatment compared with Catholics (aOR = 4.6; 95% CI = 1.02–20.8; *P* = 0.05). Similarly, being an Adventist was strongly associated with higher treatment adherence for fever (aOR = 13.42; 95% CI = 1.45–125; *P* = 0.02).

**Table 4:** Factors associated with treatment adherence for fever

Variable	Yes	No	Unadjusted			Adjusted		
			cOR	95% CI	*P*-Value	aOR	95% CI	*P*-Value
Age, years								
Younger than 15	3 (27.3)	8 (72.7)	–	–	Reference	–	–	Reference
15 and older	34 (34.7)	64 (65.3)	0.70	0.18–2.8	0.62	0.60	0.08–4.38	0.61
Gender								
Male	18 (33.3)	36 (66.7)	–	–	Reference	–	–	Reference
Female	19 (34.6)	36 (65.5)	0.95	0.43–2.1	0.89	0.68	0.23–2.00	0.49
Occupation								
Farmer	18 (30.0)	42 (70.0)	–	–	Reference	–	–	Reference
Local leader	1 (33.3)	2 (66.7)	0.86	0.07–10.1	0.90	0.58	0.01–27.60	0.78
Small trade	11 (52.4)	10 (47.6)	0.39	0.14–1.1	0.07	0.18	0.03–1.20	0.08
Vocational work	7 (28.0)	18 (72.0)	1.10	0.39–3.1	0.85	1.71	0.33–8.82	0.52
Religion								
Catholic	17 (37.8)	28 (62.2)	–	–	Reference	–	–	Reference
Protestant	10 (33.3)	20 (66.7)	1.21	0.46–3.2	0.70	5.68	0.78–41.39	0.086
Adventist	4 (28.6)	10 (71.4)	1.52	0.41–5.6	0.53	6.50	0.02–23.93	0.534
Evangelical	6 (30.0)	14 (70)	1.41	0.45–4.4	0.55	2.25	0.44–11.37	0.323
Education								
None	0 (0)	10 (100)	–	–	Reference	–	–	Reference
Primary	22 (41.5)	31 (58.5)	0.35	0.04–3.8	0.36	1.40	0.09–21.18	0.804
Secondary and above	15 (32.6)	31 (67.4)	0.48	0.05–4.7	Omitted	1.522	0.10–22.79	0.762
Location								
Rural	30 (36.6)	52 (63.4)	–	–	Reference	–	–	Reference
Urban	7 (25.9)	20 (74.1)	1.64	0.62–4.4	0.31	4.16	0.39–44.64	0.239
Facility*								
Ndama	1 (33.3)	2 (66.7)	–	–	Reference	–	–	Reference
Mahama	11 (73.3)	4 (26.7)	0.18	0.01–2.6	0.21	0.068	0.00–4.433	0.208
Masaka	6 (31.6)	13 (68.4)	1.08	0.08–14.4	0.95	0.151	0.00–15.12	0.422
Tanda	8 (29.6)	19 (70.4)	1.19	0.09–15.0	0.89	0.504	0.00–24.45	0.730
Mushubati	11 (24.4)	34 (75.6)	1.55	0.13–18.7	0.73	1	–	–
ACT knowledge								
No	37 (61.7)	23 (38.3)	–	–	Reference	–	–	Reference
Yes	35 (71.4)	14 (28.6)	0.64	0.28–0.28	0.28	1.80	0.10–30.35	0.683
ACT dislike								
No	3 (75.0)	1 (25.0)	–	–	Reference	–	–	Reference
Yes	69 (65.7)	36 (34.3)	1.56	0.15–15.6	0.70	1.0	–	–
Forgot to take ACT^†^								
No	36 (34.6)	68 (65.4)	–	–	Reference	–	–	–
Yes	1 (20)	4 (80)	2.11	0.22–19.5	0.509	–	–	–
Stopped taking ACT when better^†^								
No	35 (34)	68 (66)	–	–	Reference	–	–	–
Yes	2 (33.3)	4 (66.7)	1.20	0.17–589	0.974	–	–	–
Usually complete treatment								
No	40 (59.7)	27 (40.3)	–	–	Reference	–	–	Reference
Yes	31 (75.6)	10 (24.4)	0.47	0.20–1.1	0.09	0.47	0.03–6.81	0.582
Net use								
No	71 (67.0)	35 (33.0)	–	–	Reference	–	–	Reference
Yes	1 (33.3)	2 (66.7)	4.05	0.35–46.2	0.26	1.0	–	–
Treatment source^†^								
Health center	0 (0.0)	2 (100)	–	–	Reference	–	–	Reference
Pharmacy/outlet	25 (59.5)	17 (40.9)	0.29	0.03–2.7	0.28	0.50	0.04–7.15	0.61
CHWs	42 (71.2)	17 (28.8)	0.49	0.05–4.5	0.53	0.14	0.01–2.83	0.17
Self-treatment	5 (83.3)	1 (16.7)	–	–	–	–	–	–

ACT = artemisinin-based combination therapy; aOR = adjusted odds ratio; CHW = community health worker; cOR = crude odds ratio.

* Gishubi was not included in this analysis as no patients from Gishubi reported seeking care for fever.

^†^ The covariates “Forgot to take ACT,” “stopped taking ACT when better,” and the “self-treatment” category under “treatment source” were not included in the adjusted analysis as fewer than 10 participants reported these practices.

## DISCUSSION

This study assessed KAP regarding malaria treatment among febrile patients and/or their caregivers visiting six government health facilities in Rwanda. The study revealed that participants demonstrated a high level of knowledge regarding malaria symptoms (99%), transmission (81%), prevention and control (74.6%), and treatment (72%). However, despite relatively high malaria knowledge, only 46.1% (*n* = 83/180) of individuals diagnosed and treated for malaria completed the full dose of treatment within the recommended 3 days.

Patient adherence to antimalarials in Africa has been previously reported to be below 50%,^22^ and adherence to ACT ranges from 39% to 100%.^23^ To date, there is no published study on antimalarial adherence in Rwanda; however, the majority of adherence studies have taken place in neighboring countries. In Kenya, adherence to ACTs was reported to be only 29.4%.^24^ In contrast, another study conducted in Kenya revealed similar levels of patient adherence to ACTs (64.1%).^25^ In Uganda, a study revealed good adherence at 69.7%.^26^ Similarly, ACT adherence was also reported to be 69.8% in Tanzania.^27^

In the review conducted by Bruxvoort et al.,^22^ adherence was reported to be higher in studies where a diagnostic test was administered. In this study, 26.8% (*n* = 109/406) of participants received antimalarial drugs because of any fever without a confirmatory test. This is similar to observations in countries where malaria treatment based on fever alone is commonly practiced. For instance, in Uganda, 34.3% of participants younger than 5 years old took antimalarial drugs before getting tested.^28^ The study also revealed that 54.1% (*n* = 59/109) of participants acquired antimalarial drugs from outlets or pharmacies not affiliated with public health facilities. Similar trends were observed in a study on community pharmacies in Rwanda, which found that 88.5% of pharmacists were approached by patients seeking antimalarials and that 54% dispensed them without a prescription.^29^ This pattern is also evident in other African countries like Nigeria, where 46.5% of antimalarial drugs are self-distributed by patients and 35.8% are dispensed by outlets.^30^ Impressively, study findings revealed that all malaria cases, whether treated based on fever symptoms or based on a malaria diagnosis, were managed with artemether-lumefantrine (100%), regardless of the source of antimalarials. This aligns with Rwanda’s national malaria treatment guidelines, which designate artemether-lumefantrine as the primary treatment option nationwide for uncomplicated malaria.^31^ Furthermore, the strict regulatory oversight by the Rwanda Food and Drugs Authority ensures the traceability and quality control of antimalarial medicines available on the market.^32^

Malaria treatment practices are crucial to successful malaria control programs. This study highlights a paradox in the KAP regarding malaria treatment in Rwanda. Despite high levels of knowledge and positive attitudes toward malaria treatment and prevention strategies, these do not seem to translate into corresponding behaviors, particularly in terms of adherence to antimalarial treatment. This discrepancy is concerning as poor adherence to malaria treatment can potentiate the emergence of drug resistance, a significant threat in the era of developing resistance.^33^ This is particularly true for ACTs, where treatment in the face of ArtR parasites requires full dosing to achieve appropriate levels of partner drugs that can kill surviving parasites. Given the current status of artemisinin resistance in Rwanda and these findings that revealed poor adherence to ACT, there is an urgent need to study and better understand the drivers of poor adherence to ACT to identify solutions to improve medication-taking behaviors.

We found that individuals receiving treatment of fever were more likely to adhere to treatment compared with those with a malaria diagnosis (67% versus 46%, respectively). The difference observed in the two groups may be linked to the source of treatment. Participants with fever sought care from pharmacies/outlets, where as those with diagnosed malaria primarily sought treatment from CHWs. Adherence to antimalarials has been reported to be higher for patients accessing treatment at public health facilities.^23^^,^^27^ Community health workers are an extension of the health system, and treatment adherence to medications sourced from them has been reported to be high (>90%), even when adding antibiotics to the treatment plan.^34^ Conversely, adherence to antimalarial treatment sourced in the retail sector (outlets and pharmacies) was significantly lower compared with public health facilities (45% versus 76%, respectively).^35^ Adherence behaviors may be influenced by patient–provider interactions during the consultation, such as detailed instructions from the health worker or the use of visual aids.^27^^,^^36^ However, patient adherence behaviors not only are influenced by patient and health worker characteristics but also, can be influenced by larger health system constraints, such as stock outs, long wait times, and patient loads.^37^^,^^38^ Rwanda aims to expand the health workforce by 2028, offering hope that systemic barriers to care are improved; however, this study highlights the importance of ensuring that both public and private facilities are equipped to provide patients with information on the importance of treatment adherence.^39^^,^^40^

### Strengths.

This is the first study conducted in Rwanda to assess antimalarial treatment knowledge, attitudes and adherence practices. The present findings are valuable data for the NMCP and will be used as baseline information for monitoring malaria treatment practices at the community level.

### Limitations.

However, this study has a number of limitations. First, the generalizability of this study is limited because of the small number of participants involved in only six health facilities, which may not be representative of other health facilities in Rwanda or other countries. Additionally, the small sample size limited our precision and ability to detect factors significantly associated with treatment adherence. However, despite the limited scale of this study, the data generated still provide important insights into community knowledge and malaria treatment practices, and can inform future investigations measuring malaria treatment adherence. Second, adherence was measured using self-report, which can be subject to recall and social desirability bias. To minimize bias, we used standardized survey tools based on malaria indicator surveys and conducted interviews in a nonjudgmental manner. Finally, participants were asked about their most recent fever episodes in the last 6 months, however, the exact time when the event occurred was not recorded. This limitation prevents us from determining whether multiple reported cases refer to distinct episodes or potential double counting of the same illness. This lack of temporal data may introduce recall bias and affect the accuracy of our incidence estimates. However, even without precise timing data, the results provide us with important information on the estimated level of antimalarial treatment adherence. Additionally, demographic, behavioral, and knowledge factors associated with adherence can be explored to identify areas or populations that may be targeted for interventions.

## CONCLUSION

This study revealed adequate knowledge of malaria, treatment options, and existing prevention strategies in selected health facilities in Rwanda. However, knowledge of malaria treatment did not translate to treatment-taking behaviors. Poor adherence to malaria treatment (<50%) was noted across all health facilities. With the emergence of antimalarial drug resistance in Rwanda, there is an urgent need to strengthen malaria case management practices and monitor adherence to antimalarial treatment.

## Supplemental Materials

10.4269/ajtmh.25-0061 Supplemental Materials
