# Supplementary material for: Understanding Malaria Treatment Adherence in Rwanda: Implications for Artemisinin Resistance
Source: Am J Trop Med Hyg. 2025 Oct 28;113(6):1279–88. doi: 10.4269/ajtmh.25-0061 (PMC12676633; doi:10.4269/ajtmh.25-0061)
Supplement: Supplemental Materials [file tpmd250061.SD1.pdf]

Annex Three – KAP Questionnaire - Malaria   Study Number ……………………

Study Title: Knowledge, Attitudes, and Practices of Malaria Treatment in Rwanda: Cross-sectional Study

I am planning to interview at least 384 patients seeking treatment for fever in the following health facilities (Mahama, Ndama, Masaka, Tanda, Mushubati, and Gishubi), and in each health facility, I will interview 65 patients.

I. **DEMOGRAPHIC CHARACTERISTICS**					
1. Name of Health Facility	………………….				
2. Date of clinic Visit	…../…./……….				
3. Gender	1. Male	2. Female			
4. Are you here because of a fever	1. Yes	2. No	Temperature	………^{O}C	
5. Age (years)	:				
6. Occupation	1. Farmer	2. Local leader	3. Small trade	4. Vocational works	5.Other, specify:……………
7. Religion	1. Catholic	2. Protestant	3. Adventist	4. Muslim	5. Other Specify
8. Highest level of education	1. No formal education	2. Primary	3. Secondary	4. University and above	
9. Location in which you reside in	1. Rural	2. Semi-Rural	3. Urban		
10. Is your House sprayed with Insecticide (IRS)	1. Yes	2. No	3. I don't know		
11. If yes, how long ago your House was sprayed with IRS in Month	…………..				

II. **KNOWLEDGE OF MALARIA TREATMENT**					
**1. Have you ever heard of an illness called malaria?**	1. Yes	2. No	3. Not sure		
**2. Do you agree that the following are malaria's main signs or symptoms?**	**1. Strongly Disagree**	**2. Disagree**	**3. Neither disagree nor agree**	**4. Agree**	**5. Strongly agree**
a. Fever/Excessive Sweating,					
b. Feeling Cold/Chills.					
c. Headache					
d. Nausea And Vomiting					
e. Diarrhea					
f. Dizziness					
g. Loss Of Appetite					
h. Body Ache Or Joint Pain					

i. Pale Eyes					
j. Body Weakness					
k. Refusing To Eat Or Drink					
**3. Do you agree that the following are danger signs or symptoms of severe malaria?**	**1. Strongly Disagree**	**2. Disagree**	**3. Neither disagree nor agree**	**4. Agree**	**5. Strongly agree**
a. Shaking/Convulsions					
b. Vomiting Everything					
c. Confusion					
d. Lack of Blood (Severe Anemia)					
e. Difficulty Breathing					
f. Dizziness					
**4. In your opinion, do you agree that the following can cause malaria?**	**1. Strongly Disagree**	**2. Disagree**	**3. Neither disagree nor agree**	**4. Agree**	**5. Strongly agree**
a. Mosquito Bites					
b. Eating Immature Sugarcane					
c. Eating Cold Food					
d. Eating Other Dirty Food					
e. Drinking Cold Beer					
f. Getting Soaked with Rain					
g. Injections/Drugs					
h. Sharing Razors/Blades					
i. Bed Bugs					
**5. Do you agree that someone can protect themselves against malaria by doing the following measures**	**1. Strongly Disagree**	**2. Disagree**	**3. Neither disagree nor agree**	**4. Agree**	**5. Strongly agree**
a. Sleep Under A Mosquito Net					
b. Sleep Under Treated Net					
c. Use Mosquito Repellant					
d. Avoid Mosquito Bites					
e. Take Preventive Medication					

f. Spray House with Insecticide					
g. Use Mosquito Coils					
h. Cut The Grass Around the House					
i. Fill In Puddles (Stagnant Water)					
j. Keep House Surroundings Clean					
k. Burn Leaves					
l. Don't Drink Dirty Water					
m. Don't Eat Bad Food (Immature Sugarcane/Leftover Food					
**6. In your opinion, which people are most affected by malaria in your community choose one or more than one?**					
1. Children					
2. Adults					
3. Pregnant Women					
4. Older Adults					
5. Everyone					

**7. Have you ever heard or seen any messages/information about malaria? If yes go to Q8, if no or not sure go to Q11**	**1. Yes**	**2. No**	**3. Not sure**
**8. Where did you see or hear this message/information?**			
1. Government Clinic/Hospital			
2. Community Health Worker			
3. Friends/Family			
4. In My Home			
5. Drama Groups			
6. Peer Educators			
7. Community Meeting			
8. Posters/Billboards			
9. On Tv			
10. In The Newspaper			

11. On The Radio			
**9. How long ago did you see or hear recent messages provide answer in month(s)**	………..		
**10. What type of malaria messages/information did you see or hear?**			
1. Malaria Is Dangerous			
2. Malaria Can Kill			
3. Mosquitoes Spread Malaria			
4. Sleeping Under Mosquito Net Important			
5. Who Should Sleep Under Mosquito Net			
6. Seek Treatment For Fever Within 24 Hours/Promptly			
7. Importance Of House Spraying			
8. Not Plastering Walls After Spraying			
9. Environmental Sanitation Activities			
**11. Does your household have any mosquito nets ("tent") that can be used while sleeping?**	1. Yes	2. No	3. I don't know
**12. How many mosquito nets does your household have?**	…………		
**13. How many of the mosquito nets in your house are treated with Medicine?**	…………		
**14. Did you sleep under a treated mosquito net last night? If yes go Q15 if no go Q16**	1. Yes	2. No	3. I don't know
**15. How many days in the last week did you sleep under the treated net (range 0-7)**	………		
**16. Did any other household members sleep under a treated**	1. Yes	2. No	3. I don't know

**mosquito net last night? If yes go Q17 and if No go to Q19**			
**17. Who are all the people who slept under this net last night? (select all that apply)**			
1. Children Under 5			
2. Other Children			
3. Pregnant Woman			
4. Elderly			
5. Other (Specify			
**18. In general, how often do your under-five children sleep under a mosquito net?**	1. Always	2. Sometimes	3. Never
**19. Does your household use any other method to avoid mosquito bites? If yes go to Q20**	1. Yes	2. No	3. Not sure
**20. What other method(s) do you use specify it**	……		
III. PRACTICE MALARIA TREATMENT			
**21. Did you take Anti-malarial drugs because you were having a fever in the last six months? If yes?**	1. Yes	2. No	3. I don't know
**22. If yes the above question, where did you get those antimalarial drugs**			
1. District Hospital			
2. Health center			
3. CHWs			
4. Shops			
5. Other			
23. For how long did you take it?			
1. 1 day			
2. 2 days			
3. 3 days			
4. Above 3 days			
5. Not sure			

	Yes	No	I don't know
**24. Have you taken any medication to treat malaria in the last six months? If yes, what type of medicine did you take?**	Yes	No	I don't know
**1. Artemether-Lumefantrine (AL)**			
**2. dihydroartemisinin-*piperaquine (DAP)***			
**3. Chloroquine**			
**4. Others- Specify**			
**25. For how many days did you take this medicine?**			
1. 1 day			
2. 2 das			
3. 3 days			
4. Above 3 days			
5. Not sure			
**26. Where did you get the medication?**			
1. District Hospital			
2. Health center			
3. CHWs			
4. Shops			
5. Other			

IV. **ATTITUDE ON EXISTING MALARIA CONTROL METHODS**

**27. Attitude on existing malaria control methods**	**1. Strongly Disagree**	**2. Disagree**	**3. Neither disagree nor agree**	**4. Agree**	**5.Strongly agree**
a. Having my house sprayed is very inconvenient					
b. Chemicals used in IRS are totally safe					
c. If my house is sprayed, no need for other methods					
d. Sleeping under treated mosquito net alone does not give the guarantee of malaria prevention					

e. Mosquito net makes me feel hot when I sleep					
f. Mosquito net hinders men when doing sexual activities					
g. Mosquito net causes skin rashes and poses breathing allergies					
h. Mosquito net is good in agriculture especially for kitchen garden					
i. The quality of the mosquito net (size, shape and softness) is good					
j. If the mosquito net I own was not given to me for free, I would not buy it					
k. I prefer the mosquito net because it is free					
l. Elimination of larval breeding sources is a waste of time and very complicated					
m. Restricting and checking availability of potential breeding sources should be conducted regularly					
n. You are the one of the important people in preventing malaria Transmission					
**ATTITUDE OF MALARIA TREATMENT**					
**28. Attitude on Malaria treatment**	**1. Strongly Disagree**	**2. Disagree**	**3. Neither disagree nor agree**	**4. Agree**	**5.Strongly agree**
a. It is important to seek care for fever promptly					
b. ACTs are the nationally recommended antimalarial					

c. I do not like ACTs					
d. I always complete the full course of malaria treatment					
e. Sometimes I forget to take my malaria medicine					
f. If I am feeling better, I will stop taking my malaria medicine					

Thank you for participating in our Research
